# A Method to Estimate Health Effects Based on Error‐Prone Simulated Environmental Exposure: An Application to a Multi‐Country Study on Birthweight and Fine Particulate Matter

**DOI:** 10.1029/2025GH001789

**Published:** 2026-04-30

**Authors:** Jinting Guo, Ning Kang, Jianyu Deng, Minghao Qiu, Tao Xue

**Affiliations:** ^1^ National Health Commission Key Laboratory of Reproductive Health and Department of Epidemiology and Biostatistics Ministry of Education Key Laboratory of Epidemiology of Major Diseases (PKU) Institute of Reproductive and Child Health School of Public Health Peking University Health Science Center Beijing China; ^2^ School of Marine and Atmospheric Sciences Stony Brook University Stony Brook NY USA; ^3^ Program in Public Health Stony Brook University Stony Brook NY USA; ^4^ National Institute of Health Data Science at Peking University Institute of Medical Technology Peking University Health Science Center Beijing China

## Abstract

Earth System Models provide spatiotemporally continuous environmental exposure data but remain underused in environmental epidemiology because of uncertainty from measurement errors. We developed a novel latent‐variable approach to correct for measurement error characterized by spatiotemporal error covariance, which was derived from comparisons between Coupled Model Intercomparison Project Phase 6 (CMIP6) monthly fine particulate matter (PM_2.5_) simulations and station‐based monitoring data from 5,661 global sites. To demonstrate the utility of the framework, we associated these exposures to birthweight records from 132 Demographic and Health Surveys. The results showed variable correlations between the models and the observations (*r* = 0.40–0.68) as well as widely varying effect estimates across Earth System Models, from a 0.01 g (95% confidence interval: −0.85–0.87) reduction to a 15.11 g (12.69–17.54) reduction in birthweight per 10 μg/m^3^ increase in PM_2.5_. After correcting measurement error, the optimal estimate indicated a more precise and consistent reduction of 3.34 g (2.57–4.11) in birthweight per 10 μg/m^3^. These findings demonstrate that the negative association between PM_2.5_ exposure and birthweight is robust to different levels of measurement error embedded in CMIP6‐based exposures, and that correction for measurement error in environmental epidemiology can help avoid misestimating the effect by reducing bias and improving consistency.

## Plain Language Summary

Climate models are frequently used to simulate environmental conditions, such as air pollution, for health research, but these computer simulations often contain discrepancies when compared to real‐world measurements. This study introduces a novel statistical approach to identify and correct these “measurement errors” by comparing simulation data with observations from thousands of ground‐level monitoring stations worldwide. We used this method to analyze the impact of fine particulate matter (PM_2.5_) on the birthweight of newborns and found that different climate models produced vastly different results without correcting for errors. However, after applying the correction method, the results became consistent, confirming that exposure to higher levels of PM_2.5_ results in lower birthweights. This new method enables researchers to evaluate environmental health risks more accurately using climate model data.

## Key Points

- A framework was developed to characterize and correct spatiotemporal measurement errors in exposure
- The negative association between PM_2.5_ exposure and birthweight was consistent across CMIP6 simulations
- Correction for measurement errors yielded more precise and consistent estimates

## 1 Introduction

Simulations by Earth System Models (ESMs) are of interest for studies on health effects of climate or other environmental exposures, such as non‐optimal temperature, air pollution, and wildfires. The simulations can generate spatiotemporally continuous fields, under both the real‐world conditions and counterfactual scenarios, for exposure assessment. Take the commonly used simulations of temperature and fine particulate matter (PM_2.5_) as examples. First, they have been widely used to project the health impacts of climate change, given future socioeconomic shared pathway and climate forcing scenarios. For instance, recent research has utilized CMIP6 outputs to project global PM_2.5_ concentrations and associated premature mortality from 2021 to 2100 under different Shared Socioeconomic Pathways (SSPs) climate scenarios. The study highlights that future health burdens are highly sensitive to the chosen mitigation pathways, with significant projected variations in PM_2.5_‐related mortality across different scenarios (Chen et al., 2023). Second, ESM simulations have been utilized to identify driving forces such as a specific emission sector or a well‐recognized climate pattern (e.g., ENSO), underlying historical environmental changes and the attributable burden of diseases. For instance, one study used the Community Earth System Model (CESM) to identify increases in the intensity of heatwaves and wildfires driven by ENSO in a warming climate, which emphasized the role of land‐atmosphere feedbacks in the projected amplification of these extremes (Fasullo et al., 2018). Another study used CESM to examine the responses of PM_2.5_ to further climate, land use, and emission changes under different SSPs. The results highlighted that biomass burning and anthropogenic emissions are the main drivers of surface PM_2.5_ across all SSPs, with significant regional variations (Bhattarai et al., 2024). Third, the ESMs can also generate counterfactual scenarios of controlled exposure to reveal a causal effect on human health. For instance, a modeling study used the CESM and the Coupled Model version 3 (CM3) to simulate further changes in climate and concentrations of ozone (O_3_) and PM_2.5_ over the 21st century under a high‐greenhouse gas scenario. The findings suggested that reducing air pollution emissions could mitigate, but not prevent, the increase in mortality associated with air pollution induced by climate change. Furthermore, ESM simulations have shown great potential to assess exposures such as wildfire smoke and dust particles, which have been insufficiently measured by ground‐surface monitoring networks. For instance, a recent study incorporated a deep learning‐based fire model (DL‐fire) into the Jena Scheme for Biosphere‐Atmosphere Coupling in Hamburg version 4 (JSBACH4) land surface model within the Icosahedral Non‐hydrostatic Earth System Model (ICON‐ESM) framework. This hybrid modeling approach improved the simulation of global burned area fractions, achieving a monthly correlation of 0.8 with observed data during the evaluation period (2011–2015). The integration demonstrated the potential of combining machine learning with ESMs to enhance wildfire prediction capabilities (Son et al., 2024). The Chinese Academy of Sciences ESM version 2 (CAS‐ESM2) has been applied to simulate global dust cycles and specific dust events in East Asia. By implementing advanced dust emission parameterizations, the model effectively captured the intensity and duration of dust storms. Such simulations are crucial for regions like East Asia, where dust events can have significant health and environmental impacts (Wu et al., 2021).

Among multiple ESM products, the Coupled Model Intercomparison Project (CMIP) data have been popularly used to understand the health effects of climate and environmental changes, due to their transparency, public accessibility, organized structure, good maintenance and richness. CMIP is an international climate modeling project under the World Climate Research Programme (Meehl et al., 1997). It has become one of the foundational elements of climate science by coordinating the development and dissemination of global ESM simulations across historical, contemporary, and future climate scenarios (Eyring et al., 2016). Particularly, multiple variant simulations are documented for each ESM. Over five successive phases, CMIP has evolved into a pivotal framework for international multi‐model climate research, driving transformative advances in climate science and serving as a critical input for global climate assessments (Meehl et al., 2000, 2007; Taylor et al., 2012). As the latest phase of this framework, CMIP6 provides an updated ensemble of standardized simulations that serve as critical benchmarks for evaluating model performance and understanding global environmental changes (Eyring et al., 2016).

Like other ESM simulations, despite their rigorous design, CMIP6‐based exposures have rarely been associated with health outcomes in historical epidemiological studies, which further lowers the validity of their usage in health impact assessments or projections. A primary barrier lies in the measurement error inherent in using ESM simulations as exposure surrogates (Deser et al., 2012). Conceptually, the measurement error originates from two distinct sources. First, prediction error arises from the inherent limitations of ESMs, which rely on simplified parameterizations of complex atmospheric processes, leading to discrepancies between simulated grid‐cell concentrations and actual ambient conditions. Second, exposure allocation errors stem from spatial mismatches between the coarse resolution of ESM grid cells and the fine‐scale variations of individual exposures. Collectively, these two sources widen the gap between predicted concentrations and actual exposure levels. If ignored, the measurement error can significantly impact statistical inference on health effects in general (R. Carroll et al., 2006; Gustafson, 2003; Kipnis et al., 2003; Prentice, 1996; Shalabh, 2011; White et al., 2008). The effect size can either be overestimated or underestimated. Even the direction of the association can be reversed due to the ignorance of measurement error. Specifically, for CMIP simulations, various ESM simulations can introduce different levels of measurement error. Therefore, CMIP6 can provide an opportunity to explore to what extent different levels of ESM error influence the estimated effects on health. Moreover, ESMs remain indispensable for their exclusive ability to project future trajectories and simulate counterfactual scenarios, and the credibility of these projections also depends on the assumption that the models accurately represent real‐world exposure levels. Historical CMIP6 can be utilized as a critical validation test to characterize error structures and demonstrate the efficacy of correction methods for future applications.

What's more, since the measurement error cannot be avoided completely, in the past decade, there has been a substantial body of studies focused on correcting effect estimates given error‐prone exposure. The proposed methods included regression calibration (R. J. Carroll & and Stefanski, 1990; Gleser, 1990), simulation‐extrapolation (SIMEX) (Cook & Stefanski, 1994), instrumental variables (R. J. Carroll & Stefanski, 1994; Fuller, 1987b, 1987c; L. A. Stefanski & and Buzas, 1995), method of moments (Shalabh, 2011), multiple imputation (Cole et al., 2006), and Bayesian correction (Mallick & Gelfand, 1996; Müller & Roeder, 1997; Richardson & Gilks, 1993; Schmid & Rosner, 1993; Stephens & Dellaportas, 1992). Most traditional methods are based on the assumption of independently and identically distributed errors, failing to capture the complex error structures inherent in atmospheric models. In recent years, the field has advanced significantly beyond these simple assumptions. For instance, Bergen et al. advanced regression‐based approaches to correct for measurement error in PM_2.5_ component exposures by leveraging spatial correlations, demonstrating the importance of correcting for spatial structures in exposure prediction models to avoid biased health inferences (Bergen et al., 2013). In parallel, Bayesian frameworks have seen significant developments, offering flexible solutions for uncertainty quantification. Comess et al. introduced a flexible Bayesian kernel density estimation approach to propagate uncertainty, effectively handling non‐Gaussian posterior distributions (Comess et al., 2023). Park et al. and Lee et al. developed two‐stage Bayesian frameworks utilizing Vecchia approximations, enabling efficient error correction for large data sets (Lee et al., 2024; Park et al., 2026). These efforts have also been extended into the causal inference domain, proposing a unified method to simultaneously correct for measurement errors in both exposures and confounders (Kim, 2024). Despite these methodological breakthroughs, a critical gap remains. Many of these advanced approaches are optimized for regional domains or specific cohort studies, which fail to accommodate the globally spatiotemporally‐correlated errors embedded in ESM simulations inherently. Furthermore, existing methods mainly focus on single‐source exposure surfaces. They are less efficient at modeling the measurement error embedded in CMIP6 data, because of failure to make use of ensemble simulations from multiple ESMs and their variants.

To address these gaps, we propose an optimal estimator that explicitly quantifies and adjusts for spatiotemporally structured measurement errors in multiple CMIP6 simulations. To demonstrate its use, we apply it to associate CMIP6‐simulated PM_2.5_ concentrations with birthweight reduction. We select the exposure‐outcome pair for the following reasons. First, the association has been well identified by many previous studies, providing an expectation for our demonstrative analysis. Second, the outcome (i.e., birthweight) can be measured by a continuous dependent variable, leading to a likelihood function that can be easily incorporated into the spatiotemporal Gaussian model of measurement error. Finally, there is a publicly available data set on individual‐level birthweight, which has been utilized in our previous studies and makes the demonstrative analysis reproducible. Specifically, we established a covariance function to quantify the spatiotemporal correlation of errors, which are measured by discrepancies between CMIP6 simulations and observations. The true exposure was incorporated into a likelihood function that measures the exposure‐outcome association and measurement errors simultaneously. Additionally, our optimal estimator was also validated by a statistical simulation and compared to traditional methods.

## 2 Method

### 2.1 Data Acquisition and Preprocessing

In this study, we obtained historical PM_2.5_ concentrations from 76 CMIP6 simulations generated by 10 ESMs, namely, MIROC6, MIROC‐ES2H, GISS‐E2‐1‐H, GISS‐E2‐1‐G, GISS‐E2‐2‐G, MRI‐ESM2‐0, MIROC‐ES2L, NorESM2‐MM, MPI‐ESM‐1‐2‐HAM, and NorESM2‐LM. A detailed list is documented in Table S1 in Supporting Information S1. The monthly PM_2.5_ concentrations used in this study were obtained directly from the CMIP6 variable *mmpm2p5* where available. According to Turnock et al. (2020), this diagnostic variable represents the sum of individual aerosol component masses (BC, OA, Sulfate, Nitrate, and fine‐mode Dust/Sea‐salt) calculated by the modeling centers. We extracted monthly averages of PM_2.5_ concentration data for each grid cell, from January 2000 to December 2014.

Ground‐surface observations of PM_2.5_ concentrations were regarded as the gold standard for exposure assessment and were used to validate the simulated concentrations. We extracted daily PM_2.5_ concentrations collected by a previous study from 5,661 stations maintained by government agencies, such as the European Environmental Agency or the Environmental Protection Agencies of the United States, China, and Australia (Riley et al., 2019; Yu et al., 2023). To ensure temporal consistency, we aggregated the original records by months and restricted the time series to the period from 2000 to 2014. If there were ≤20 valid daily measurements within a month for a certain station, the aggregated value was assigned as missing. Each monthly observation was paired with 76 simulations extracted from the CMIP6 database by timestamp (year‐month) and spatial grid. Specifically, we extracted the corresponding simulated PM_2.5_ concentrations for each individual monitoring station using bilinear interpolation resampling based on the station's specific geographic coordinates.

Individual‐level birthweight records and covariates were obtained from the 132 Demographic and Health Surveys (DHS). Each of them is nationally representative, collected by a stratified two‐stage cluster sampling design. Details of the DHS data set are presented in our previous studies (Lu et al., 2025; Tong et al., 2024; Xue et al., 2023). The health outcome is birthweight. The covariates include infant sex (female or male), singleton or not, cesarean section or not, maternal age, maternal education attainment, maternal body mass index (≤18.5, >18.5 to 25.0, >25.0 to 30.0, or >30.0 kg/m^2^), marital status (divorced, living with partner, married, never in union, separated, or widowed), antenatal care attendance or not, urban or rural residence, parity (primipara or multipara), sex of household head (female or male), medical insurance coverage or not, household size, cooking fuel type (agricultural crop, animal dung, biogas, charcoal, coal, electricity, kerosene, liquefied natural gas, natural gas, wood, or others), source of drinking water (bottled water, natural water, piped water, rain water, tank water, tube water, well water, or others), and type of toilet facility (composting, flush, or none). Individual records from January 2000 to December 2014 were filtered to exclude observations with missing birthweight or geographic information. Missing covariates were subsequently imputed via multiple imputation by chained equations.

In the following analysis, we used a method of spatiotemporal epidemiology to evaluate the association between birthweight and PM_2.5_ exposure. All CMIP6 simulations were regridded to a standardized spatial resolution of 2.5° × 2.0° using bilinear interpolation. To match the CMIP6 simulations, we first assigned each individual to the cell of the 2.5° × 2.0° grid, and further aligned them temporally by birth month. To minimize the influence of stochastic variability and outliers inherent in small sample sizes and to ensure robust statistical inference, if there were <10 valid individual records within a spatiotemporal unit (grid/month), those records were excluded. A random forest regression model was constructed to predict birthweight from all individual‐level covariates (excluding PM_2.5_ concentrations). Specifically, two hyper‐parameters, the number of trees (*ntree*) and the number of variables to split at each node (*mtry*), were optimized through grid searches, with *ntree* ranging from 400 to 2,000 in increments of 100 and *mtry* ranging from 6 to 10 in increments of 1. The performance of the model was evaluated by root mean square error (RMSE) in 10‐fold cross‐validation, as shown in Figure S1 in Supporting Information S1. For each individual, an expected birthweight was calculated as the random forest prediction, and its discrepancy from the observed birthweight was derived as the birthweight anomaly. Finally, the average of all individual‐level anomalies within a spatiotemporal unit was derived as the outcome variable to be linked to PM_2.5_ concentrations.

### 2.2 Spatiotemporal Gaussian Model of PM_2.5_ Measurement Error

To model the measurement error embedded in the simulated PM_2.5_ concentrations by the *i*th CMIP6 product (***X***_*i*_), we assumed a Gaussian spatiotemporal model as follows:

$$X_{i}\;∼\;\text{Gau}\;\left(a_{i}\mu +b_{i},\Sigma \;\left(h,u\left|\theta )),\Sigma \;\left(j,k\right)=C\;(h_{j,k},u_{j,k}\right|\theta \right.\right)$$

where ***X***_*i*_ denotes a vector of error‐prone *i*th‐model simulations paired with the error‐free values (***μ***); *a*_*i*_ and *b*_*i*_ respectively denote the simulation‐specific slope and intercept for calibration; **Σ** denotes a variance‐covariance matrix; its element **Σ**(*j, k*) is parameterized by the covariance function C (˖| ***θ***); *h*_*j,k*_ and *u*_*j,k*_ denote the corresponding spatial and temporal distance, respectively. We used the ground‐surface observations as the error‐free values (***μ***), and fit the covariance function based on least‐squares residuals. The empirical covariance function was quantified by computing correlation coefficients between the residuals across different combinations of spatial and temporal distances. Specifically, spatial distance (*h*) was computed by the Haversine formula, and temporal distance (*u*) was assessed at monthly intervals. The empirical covariance was calculated by multiplying the correlation coefficients by variance of the residuals. We utilized six candidate functions (Text S1 in Supporting Information S1) to fit the spatiotemporal covariance function by the non‐linear least squares (NLS) method. (Bevilacqua et al., 2010; Gneiting, 2002; Gneiting et al., 2006). The selection of the optimal function was determined based on minimizing the residual standard error derived from the NLS models. To derive stable estimates, we assumed a uniform covariance function for all simulations from different ESMs and their variants. Finally, the optimal covariance function was estimated as follows:

$$C\left(h,u\right)=159.6\frac{e^{{-\left(\frac{h}{1,952}\right)}^{0.568}{\left[1+{\left(\frac{u}{3.258}\right)}^{0.670}\right]}^{0.284}}}{{\left[1+{\left(\frac{u}{3.258}\right)}^{0.670}\right]}^{1.5}}.$$

Alternatively, to evaluate the robustness of the assumption of a shared error structure, we performed a sensitivity analysis by fitting ESM‐specific covariance functions.

### 2.3 Association Model With Measurement Error Calibration

To associate birthweight with PM_2.5_ exposure during late‐term gestation, we first employed several traditional methods as comparisons. Levels of exposure were approximated by PM_2.5_ concentrations at the month of delivery. These methods included (a) the simulation‐specific model that associates birthweight anomaly with concentrations simulated by a certain ESM, (b) the average‐exposure model that associates birthweight anomaly with the average of all simulations, and (c) the average‐effect model that pools all simulation‐specific associations together by a random‐effect meta‐analysis. The average‐effect model is also known as a two‐stage model.

The optimal estimator with calibrated measurement errors was developed by combining the association model and the spatiotemporal Gaussian model of measurement errors in a likelihood function. The association model links birthweight anomaly with a latent variable to reflect the error‐free exposure (***μ***), which was further linked to all simulations by the measurement error model. Regression coefficients and the latent exposure variable were iteratively estimated using the maximum likelihood approach. The validity of the optimization procedure was confirmed through the negative definiteness of the Hessian matrix (Text S2 in Supporting Information S1). Uncertainty was quantified using Fisher information matrices, with 95% confidence intervals (CIs) derived from asymptotic normality.

We performed a sensitivity analysis to compare the three models that evaluate the health effect based on multiple exposures, namely, (a) our optimal estimator with calibration of measurement error, (b) the average‐exposure model, and (c) the average‐effect model. The three models were applied within a subset of ESM‐specific simulations (i.e., the simulations of different variants generated by the same ESM). To ensure enough statistical power, we selected only ESMs with ≥10 simulation variants. Therefore, we selected four ESMs that were GISS‐E2‐1‐G, MIROC6, MIROC‐ES2L, and MRI‐ESM2‐0. A better model was expected to generate more consistent estimates across different ESM‐specific subsets.

To further validate the optimal estimator, we conducted a statistical simulation of 500 iterations. The estimated parameters from the previous analysis were utilized as the pseudo truth to generate data in the statistical simulation. In each iteration, we randomly sampled 6,000 grid cells as the surveyed locations, and introduced measurement errors using the corrected parameters. The health effect was evaluated by three models. Performance of the three models was evaluated by comparing their estimates to the pseudo‐truth.

All analyses were conducted using R software (version 4.4.3), with the packages “geosphere,” “caret,” and “ranger.”

## 3 Results

**Figure 1:**
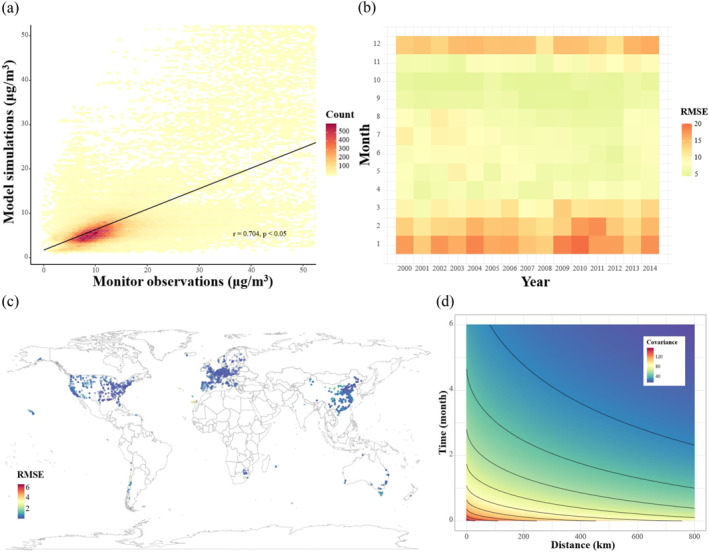
Characterization of measurement error in model simulations of PM_2.5_. (a) Correlations between the average of all selected simulations and monitoring observations; (b) Temporal distribution of measurement error; (c) Spatial distribution of measurement error; (d) Spatiotemporal covariance function of measurement error based on an ensemble of all simulations.

The simulated concentrations show moderately good agreement with the ground‐surface observations of PM_2.5_, but the magnitude of agreement varies between ESMs and their variants, showing a heterogeneous measurement error. All simulations exhibit positive correlations, with correlation coefficients ranging from 0.40 (MIROC‐ES2L r24i1p1f2) to 0.68 (MIROC6 r7i1p1f1) (Table S2 in Supporting Information S1). Averaging all simulations further improves the correlation coefficient to 0.70 (Figure 1a). For the four ESMs used in the following sensitivity analysis, the correlation coefficients are 0.59 (GISS‐E2‐1‐G), 0.66 (MIROC6), 0.51 (MIROC‐ES2L), and 0.61 (MRI‐ESM2‐0). Detailed scatterplots for the four models are shown in Figure S2 in Supporting Information S1. Temporally, RMSE values show a seasonal pattern, characterized by higher RMSE in winter and lower RMSE in summer, which is generally stable from 2000 to 2014 (Figure 1b). Spatial distribution of measurement errors is shown at monitoring stations (Figure 1c). In North America, the eastern and western coastal regions show relatively lower RMSE values, while the central regions exhibit moderate RMSE. Across Europe, the RMSE values are relatively low. In East Asia, most stations show low RMSE values, except for a few in North China. For the rest of the regions such as Africa and Latin America, monitoring stations were sparsely distributed and the corresponding RMSE values are relatively high, suggesting limited accuracy of the simulations. The errors were found to be spatiotemporally autocorrelated. Based on the ensemble of all errors, the estimated covariance function is shown in Figure 1d. It decreases rapidly with increasing temporal lag. A sensitivity analysis further showed that the covariance function did not vary considerably across different ESMs (Figure S3 in Supporting Information S1).

**Figure 2:**
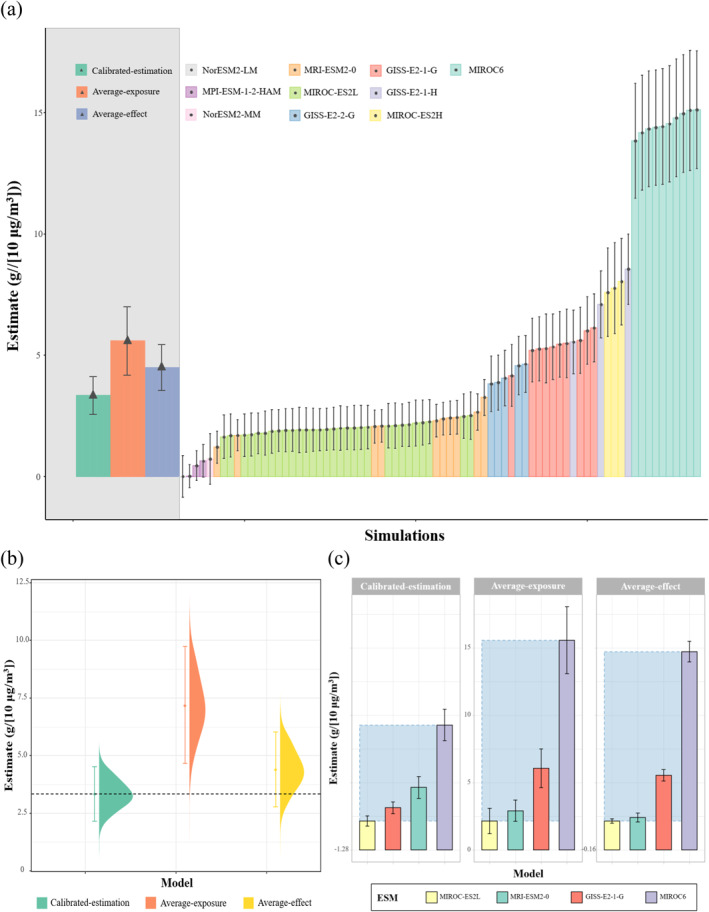
Simulation‐derived estimates of PM_2.5_ exposure effects on birthweight. (a) Estimates of the birthweight reduction for a per‐10‐μg/m^3^ increment in PM_2.5_ exposure derived from selected simulations. Shaded backgrounds denote the estimates from the ensemble of all simulations; other bars are estimates from individual simulations. (b) Method validation with effect estimates from three models: the calibrated‐estimation model, the average‐exposure model, and the average‐effect model, with the pre‐specified true effect indicated by a dashed line. (c) ESM‐specific ensemble estimates from three models: the calibrated‐estimation model, the average‐exposure model, and the average‐effect model.

An increment in PM_2.5_ concentration was associated with a reduction in birthweight by multiple conventional methods that do not account for measurement error, including traditional models relying on individual simulations, average‐exposure models, and average‐effect models. The size of the estimated effects differed, which might be caused by heterogeneous measurement error. First, effect estimates exhibited notable discrepancies across different ESMs or their variants (Figure 2a). For a per‐10‐μg/m^3^ increment in PM_2.5_ exposure during the late‐term gestation, the estimated reduction in birthweight varied from 0.01 g (−0.85–0.87, by NorESM2‐MM r1i1p1f1) to 15.11 g (12.69–17.54, by MIROC6 r6i1p1f1). The difference between the two effect estimates was recognized as statistically significant by a Wald test (*P* value <0.05). Simulations from different variants of the same model showed comparable effect estimates. Second, based on the average of all simulations, the effect on the birthweight reduction for a per‐10‐μg/m^3^ increment in PM_2.5_ exposure was re‐estimated as 5.59 g (4.18–7.00). Third, based on the meta‐analysis of all simulation‐specific estimates, the pooled effect was 4.49 g (3.56–5.43), which was not significantly different from the result of the average‐exposure model (Wald test *P* value = 0.21).

After calibrating the measurement error, the birthweight reduction for a per‐10‐μg/m^3^ increment in PM_2.5_ exposure during the late‐term gestation was re‐estimated as 3.34 g (2.57–4.11), slightly lower than that from either average‐exposure or average‐effect model. Statistical simulation showed that the error‐calibrated optimal estimator outperformed the previous two models (Figure 2b), by generating an unbiased estimate. In contrast, both average‐exposure and average‐effect models overestimated the effect by 67.2% (10.0–124.4) and 34.4% (−7.3–76.2), respectively. Since heterogeneity in measurement errors could potentially cause inconsistency in estimated effects, calibrating those errors was found to improve the stability. In the absence of correction, using either average‐exposure or average‐effect models, the estimates derived from four specific ESM subsets were widely dispersed, with the range of estimates enlarged to 13.4 g (10.0–16.8) and 12.6 g (11.6–13.5), respectively (Figure 2c). In contrast, applying the optimal estimator significantly improved consistency. The corrected birthweight reductions for these specific ESMs, namely, MIROC‐ES2L, GISS‐E2‐1‐G, MRI‐ESM2‐0, and MIROC6, were 0.87 g (0.49–1.24), 1.84 g (1.42–2.27), 3.35 g (2.54–4.17), and 7.98 g (6.81–9.15), respectively. Consequently, the range of effect estimates was substantially narrowed to 7.11 g (5.57–8.66). Our results demonstrate that calibrating measurement error in environmental exposure can effectively mitigate the structural discrepancies between models, reducing bias and increasing robustness of health effect estimates.

## 4 Discussion

This study presents a novel framework for correcting measurement error in exposure data sets, a strategy that can be broadly applied to any data source with intrinsic uncertainties. To our knowledge, this is the first study to conduct formal measurement error correction using globally available air pollution predictions regarding spatiotemporal correlation, filling a critical gap left by prior studies that largely focused on regional analyses or specific cohorts. Our findings demonstrate the importance of adjusting for measurement error to generate unbiased and robust estimates of the health effects of environmental exposures evaluated by ESMs and their extensions. Our study, once again, confirms the association between PM_2.5_ exposure and birthweight reduction. We further show that the direction of the association is robust given different levels of measurement error, enhancing the strength of evidence on the PM_2.5_‐induced birthweight reduction.

In this study, we selected CMIP6 simulations as the target for the measurement error characterization and correction, rather than the satellite‐based or data‐fusion products that have been widely used in previous epidemiological studies. Our choice is grounded in the following three reasons. First, ESM simulations are still underused in studies because of the known presence of measurement error. The unique ensemble property of CMIP6, comprising multiple models and numerous variant simulations, provides the substantial data volume necessary for a rigorous characterization of measurement error. And the discrepancies inherent in ESMs create an environment, in which we can demonstrate that our latent‐variable approach can effectively harmonize diverse and error‐prone inputs. Second, ESMs generate spatiotemporally continuous fields for a broad spectrum of environmental variables, such as specific particulate components, dust, and temperature, which often lack global, observation‐constrained counterparts. Validating our framework on PM_2.5_ establishes a protocol that can be extended to these other variables where fusion data are unavailable. Last, CMIP6 provides the basis for projecting future health impacts under SSPs. Calibrating historical simulations against observations is essential for minimizing uncertainty in future risk assessments, a goal that historical satellite data cannot achieve.

To provide a comprehensive understanding of the reliability of our estimates, it is important to distinguish between three sources of uncertainty: statistical variability, exposure data choice, and model structure. First, statistical uncertainty, representing random sampling errors, was quantified using 95% CIs derived from the Fisher information matrix. Second, uncertainty regarding exposure data choice stems from the structural differences among ESMs. As shown in our results, uncorrected effect estimates varied widely across different ESMs. However, our method demonstrated robustness by narrowing this range and producing consistent estimates across varying simulation inputs. Third, uncertainty related to model structure arises from assumptions made in the measurement error framework, specifically the spatiotemporal Gaussian assumption and the selection of the covariance function. While we optimized the covariance function based on goodness‐of‐fit and validated it through sensitivity analyses, we acknowledge that alternative structural assumptions could influence the characterization of error patterns.

Methods to correct for measurement error are rarely applied in epidemiologic research (Jurek et al., 2006; Shaw et al., 2018). As it is commonly thought that the effect of measurement error is to bias estimates of exposure effects toward the null, the measurement error would be ignored when testing the null hypothesis of no effect (R. J. Carroll, 2014). But the true effect size would always be underestimated if measurement error is ignored (J. Hausman, 2001; J. A. Hausman et al., 1995). Therefore, correcting for measurement error is critical to avoid biased inferences, especially in complex modeling frameworks. Despite these theoretical advances, practical implementation remains challenging due to the limited availability of validated methods and accessible software tools (Keogh et al., 2020). Our study provides a correction framework for spatiotemporal error structure, thereby enhancing analytical capabilities in environmental epidemiological applications.

In order to select an appropriate measurement error correction strategy for CMIP6, it is necessary to critically evaluate the trade‐offs inherent in existing methodologies. Several approaches have been developed in recent decades. Among these, regression calibration is the most commonly used empirical approach. The basic principle of regression calibration is to replace true exposures with the regression of true exposures on error‐prone exposure measures and other covariates, a procedure called the calibration function (R. J. Carroll, 2014). Its relative simplicity and intuitive appeal make it widely applicable in a variety of models, including linear (R. J. Carroll & Stefanski, 1990; Gleser, 1990), proportional hazard (Prentice, 1982), generalized linear (Armstrong, 1985; Fuller, 1987a) and logistic (Rosner et al., 1989, 1990) models. However, while it performs well in scenarios where the calibration function is well specified, its accuracy diminishes when error models deviate from the classical additive assumptions (R. Carroll et al., 2006; Shalabh, 2011). SIMEX, sharing the simplicity of regression calibration, is a simulation‐based method. It addresses measurement error by simulating additional error variance and extrapolating results to an error‐free scenario, and provides intuitive graphical diagnostics (Cook & Stefanski, 1994; L. A. Stefanski & Cook, 1995). However, it poses a burden on computational resources and requires sensitivity analyses to validate the extrapolation procedure. Particularly, its performance is highly sensitive to the choice of extrapolation model (R. Carroll et al., 2006). Instrumental variables methods address measurement error through the use of external variables that are correlated with true exposure but independent of the measurement error (Fuller, 1987b). This approach avoids the need for direct estimation of the measurement error variance, making it useful when replicates or validation data are unavailable (R. J. Carroll, 2014). However, the methods also face challenges, including the difficulty of identifying valid instruments and the risk of bias due to weak instruments (Andrews et al., 2019). Method of moments avoids distributional assumptions by estimating parameters through moment equations (Stefanski & Boos, 2002), but it is not well suited for inferring high‐dimensional parameters (R. Carroll et al., 2006). Multiple imputation treats measurement error as a missing data problem, and offers flexibility under missing at random assumptions (Cole et al., 2006). However, it may risk propagating bias if the imputation model inadequately captures the mechanism underlying the error generation (Little & Rubin, 2019). Maximum‐likelihood‐based methods are appealing for the measurement error correction, due to their flexibility. When the outcome model and exposure error model can be specified simultaneously, the maximum‐likelihood method provides a generalizable framework to calibrate the measurement error (R. J. Carroll, 2014). Particularly, Bayesian models can be considered an extension of likelihood models. They start with the likelihood function and add prior probability distributions for all unknown parameters to the function. This study selected a maximum‐likelihood parametric model because the ensemble property of the CMIP6 database provides a unique opportunity to characterize measurement error in detail. The method is advantageous in adapting the spatiotemporally autocorrelated model, which is suitable for characterizing errors embedded in environmental data. To reduce computational complexity, a two‐step estimation strategy was designed, leaving only the target parameter for iterative optimization. Notably, this approach allows for a theoretical extension to include additional covariates in the full likelihood estimation. While this study assumed fixed true exposure, an alternative specification could include distributional assumptions for true exposure. As mentioned above, our model can be easily modified under a Bayesian framework, which is commonly used in spatiotemporal statistics as well.

Although various methods to calibrate measurement errors have different strengths and limitations, only a small number of studies have compared their performances in specific settings. Messer et al. compared three approaches, namely regression calibration, multiple imputation, and maximum likelihood, applied to logistic regression. They found that maximum likelihood outperformed the other two approaches given a large sample size (Messer & Natarajan, 2008). Thoresen et al. also compared maximum likelihood and regression calibration in a simulation study based on the logistic model. They found both performed well, but that regression calibration offered computational convenience (Thoresen & Laake, 2000). Cole et al. compared regression calibration to multiple imputation in a Cox proportional hazards model for survival analysis. They found that the performance of multiple imputation calibration depended on the sample size (Cole et al., 2006). Therefore, the choice among these methods depends largely on performance, computational burden, and the objectives of the analysis. Future studies are needed to develop advanced methods specifically focusing on ESM simulations, like CMIP6.

Certain limitations in this study should be mentioned. First, the accuracy of the covariance function depends on the representativeness of the validation data set. Although ground monitoring stations provided reference measurements, their uneven global distribution creates a spatial mismatch with the coverage of health records. Our method extrapolates the error covariance structure from observation‐rich regions to regions with sparse or no monitoring data, which is a strong assumption given known regional differences in emission sources, aerosol composition, and atmospheric process. However, CMIP6 simulations are generated based on physical and chemical principles rather than ground monitoring data, which satellite‐based, data‐fusion, or machine‐learning products heavily rely on. The generation process is independent of the location or density of ground monitoring stations. And the correlation in spatiotemporal covariance is largely driven by the atmospheric transport mechanisms and the spatial resolution of the models, both of which are globally applicable physical properties. Therefore, it is reasonable to estimate the covariance parameters from observation‐rich regions and transfer this structure to regions with sparse or no observations. Second, spatiotemporal covariance modeling requires significant high‐quality validation data, which may not always be feasible for studies with limited data sets. Third, partitioning the study area into fixed grids and performing parameter estimation at this aggregated level may compromise exposure assessment accuracy. This approach simplifies the spatial variability of PM_2.5_, potentially introducing misclassification in regions with high heterogeneity. However, it is important to note that the proposed characterization and correction framework can be generalized in terms of methodology. In theory, it can be applied directly to finer grids or to all individual‐level samples to enhance precision. The choice of the current grid size is to balance methodological rigor with practical implementation. Finally, the maximum likelihood estimation method employed here introduces computational complexity that requires advanced optimization algorithms. While advances in high‐performance computing have mitigated limitations related to data storage, the computational cost of applying complex error‐correction algorithms to global ensembles remains a critical constraint. Specifically, our optimal estimator requires extensive matrix operations. Conducting this analysis at the native resolution of every model proved computationally prohibitive. Therefore, we addressed this trade‐off between precision and feasibility by regridding simulations to a standardized resolution (2.5° × 2.0°). This approach significantly reduced the computational burden, enabling the efficient implementation of our rigorous calibration framework while preserving essential regional spatial patterns. The proposed method provides a novel framework, while these limitations highlight directions for improvement, particularly in terms of scalability and parameter estimation efficiency.

## 5 Conclusions

The negative association between PM_2.5_ and birthweight is robust, while PM_2.5_ simulations derived from CMIP6 models exhibit non‐negligible measurement error at various levels when compared with ground monitoring data. By developing and applying a novel latent‐variable approach that accounts for spatiotemporally structured classical measurement error, the estimated effect showed improved consistency. Further research could extend this method to other environmental exposures and health outcomes, as well as improve the efficiency of parameter estimation.

## Conflict of Interest

The authors declare no conflicts of interest relevant to this study.

## Supporting information

Supporting Information S1

## Data Availability

All data are publicly available. The health data were obtained from the Demographic and Health Surveys (DHS) Program. Due to the large number of surveys, a complete list of the specific countries, survey years, and individual data set identifiers is provided in Table S3 in Supporting Information S1. These data can be freely downloaded from https://dhsprogram.com/data/available‐datasets.cfm following registration and by selecting the specific surveys listed in Table S3 in Supporting Information S1. The ground‐surface observations of PM_2.5_ are accessible at (Xu et al., 2025). The CMIP6 data sets were retrieved from the Earth System Grid Federation (ESGF) portal, and this study included historical PM_2.5_ simulations generated by 10 ESMs, namely, MIROC6, MIROC‐ES2H, GISS‐E2‐1‐H, GISS‐E2‐1‐G, GISS‐E2‐2‐G, MRI‐ESM2‐0, MIROC‐ES2L, NorESM2‐MM, MPI‐ESM‐1‐2‐HAM, and NorESM2‐LM (Bentsen et al., 2019; Hajima et al., 2019; Nasa‐GISS, 2018, 2019a, 2019b; Neubauer et al., 2019; Seland et al., 2019; Tatebe & Watanabe, 2018; Watanabe et al., 2021; Yukimoto et al., 2019).
