# Supplementary material for: A Method to Estimate Health Effects Based on Error‐Prone Simulated Environmental Exposure: An Application to a Multi‐Country Study on Birthweight and Fine Particulate Matter
Source: Geohealth. 2026 Apr 30;10(5):e2025GH001789. doi: 10.1029/2025GH001789 (PMC13130145; doi:10.1029/2025GH001789)
Supplement: Supplementary file 1 — Supporting Information S1 [file GH2-10-e2025GH001789-s001.pdf]

Supporting Information for

A method to estimate health effects based on error-prone simulated environmental exposure: an application to a multi-country study on birthweight and fine particulate matter

Jinting Guo[1], Ning Kang[1], Jianyu Deng[1], Minghao Qiu[2, 3], Tao Xue[1, 4 #]

1. Institute of Reproductive and Child Health, National Health Commission Key Laboratory of Reproductive Health and Department of Epidemiology and Biostatistics, Ministry of Education Key Laboratory of Epidemiology of Major Diseases (PKU), School of Public Health, Peking University Health Science Center, Beijing 100191, China
2. School of Marine and Atmospheric Sciences, Stony Brook University, Stony Brook, NY, USA
3. Program in Public Health, Stony Brook University, Stony Brook, NY, USA
4. State Environmental Protection Key Laboratory of Atmospheric Exposure and Health Risk Management, Center for Environment and Health, Peking University, Beijing 100871, China

[#]Corresponding to
Tao Xue (txue@hsc.pku.edu.cn)

Contents of this file

Text S1 to S2
Figures S1 to S3
Tables S1 to S3

Text S1. We included six spatiotemporal correlation models in this study. In the following formulas, h and u represents the spatial and temporal distance, repectively.

Non-separable models:

1. gneiting model:

$$R(h,u) = \frac{e^{\frac{-h^{\nu}}{(1+u^{\lambda})^{0.5\gamma\nu}}}}{1+u^{\lambda}}$$

The parameters ν and λ take values in $[0,2]$; the parameter γ take values in $[0,1]$.

2. gneiting_GC model:

$$R(h,u) = \frac{e^{\frac{-u^{\lambda}}{(1+h^{\nu})^{0.5\gamma\lambda}}}}{1+h^{\nu}}$$

3. iacocesare model:

$$R(h,u) = (1+h^{\nu}+u^{\lambda})^{-\delta}$$

The parameters ν and λ take values in $[1,2]$; the parameters δ must be greater than or equal to half the space-time dimension.

4. porcu2 model:

$$R(h,u) = \frac{e^{-h^{\nu}(1+u^{\lambda})^{0.5\gamma\nu}}}{(1+u^{\lambda})^{1.5}}$$

The parameters ν and λ take values in $[0,2]$; the parameter γ take values in $[0,1]$.

Separable models:

1. exp_exp model:

$$R(h,u) = e^{-h}e^{-u}$$

2. exp_cauchy:

$$R(h,u) = e^{-h}(1+h^2)^{-\beta}$$

The parameter β is positive.

The covariance model is

$$C(h,u) = var \times R(\frac{h}{scale_s}, \frac{u}{scale_t})$$

where var is the variance parameter, and $scale_s$, $scale_t$ are the spatial and temporal scaling parameters, respectively.

Text S2. Let $\boldsymbol{Y} = (y_1, y_2, \cdots, y_t)'$ be the observed outcome, $\boldsymbol{\mu} = (\mu_1, \mu_2, \cdots, \mu_t)'$ be the true value of exposure, $\boldsymbol{X}_i = (x_{i1}, x_{i2}, \cdots, x_{it})'(i = 1, 2, \cdots, N)$ be the exposure of i th simulation, σ^2 be the variance of the measurement error in the observed outcome, and $\boldsymbol{\Sigma}$ be the covariance matrix of the measurement error in the simulated exposures. The likelihood would be

$$\begin{aligned} L =& \frac{1}{\sqrt{(2\pi)^t}\sigma^t} \exp(-\frac{1}{2\sigma^2}(\boldsymbol{Y} - \boldsymbol{\mu}\beta_1 - \boldsymbol{E}\beta_0)'(\boldsymbol{Y} - \boldsymbol{\mu}\beta_1 - \boldsymbol{E}\beta_0)) \\ & \prod_{i=1}^{n}[\frac{1}{\sqrt{(2\pi)^t}|\boldsymbol{\Sigma}|} \exp(-\frac{1}{2}(\boldsymbol{X}_i - \boldsymbol{\mu}a_i - \boldsymbol{E}b_i)'\boldsymbol{\Sigma}^{-1}(\boldsymbol{X}_i - \boldsymbol{\mu}a_i - \boldsymbol{E}b_i))] \end{aligned}$$

where a_i and b_i are slope and intercept terms of linear regression between simulated exposure and true exposure (ground-surface observation), respectively, $\boldsymbol{E} = (1, 1, \cdots, 1)'$. Ignoring additive constants and focusing on the log-likelihood, we obtain

$$\begin{aligned} \ln L =& -\frac{t}{2}\ln\sigma^2 - \frac{1}{2\sigma^2}(\boldsymbol{Y} - \boldsymbol{\mu}\beta_1 - \boldsymbol{E}\beta_0)'(\boldsymbol{Y} - \boldsymbol{\mu}\beta_1 - \boldsymbol{E}\beta_0) \\ & -\frac{1}{2}\sum_{i=1}^{N}(\boldsymbol{X}_i - \boldsymbol{\mu}a_i - \boldsymbol{E}b_i)'\boldsymbol{\Sigma}^{-1}(\boldsymbol{X}_i - \boldsymbol{\mu}a_i - \boldsymbol{E}b_i) \end{aligned}$$

Then the following equations can be obtained by partial differentiation of log-likelihood with respect to σ^2, β_1, β_0, and $\boldsymbol{\mu}$:

$$\begin{aligned} \frac{\partial \ln L}{\partial \sigma^2} &= -\frac{t}{2\sigma^2} + \frac{(\boldsymbol{Y} - \boldsymbol{\mu}\beta_1 - \boldsymbol{E}\beta_0)'(\boldsymbol{Y} - \boldsymbol{\mu}\beta_1 - \boldsymbol{E}\beta_0)}{2\sigma^4} \\ \frac{\partial \ln L}{\partial \beta_1} &= \frac{\boldsymbol{\mu}'(\boldsymbol{Y} - \boldsymbol{\mu}\beta_1 - \boldsymbol{E}\beta_0)}{\sigma^2} \\ \frac{\partial \ln L}{\partial \beta_0} &= \frac{\boldsymbol{E}'(\boldsymbol{Y} - \boldsymbol{\mu}\beta_1 - \boldsymbol{E}\beta_0)}{2\sigma^2} \\ \frac{\partial \ln L}{\partial \boldsymbol{\mu}} &= \frac{1}{\sigma^2}\beta_1(\boldsymbol{Y} - \boldsymbol{\mu}\beta_1 - \boldsymbol{E}\beta_0) + \sum_{i=1}^{n}\boldsymbol{\Sigma}^{-1}(\boldsymbol{X}_i - \boldsymbol{\mu}a_i - \boldsymbol{E}b_i) \end{aligned}$$

The equations are set to zero and the solution is the maximum likelihood estimates of σ^2, β_1, β_0 and $\boldsymbol{\mu}$ as

$$\begin{aligned} \widehat{\sigma^2} &= \frac{(\boldsymbol{Y} - \boldsymbol{\mu}\beta_1 - \boldsymbol{E}\beta_0)'(\boldsymbol{Y} - \boldsymbol{\mu}\beta_1 - \boldsymbol{E}\beta_0)}{t} \\ \widehat{\beta_0} &= \frac{\boldsymbol{E}'\boldsymbol{Y} - \boldsymbol{E}'\boldsymbol{\mu}\beta_1}{t} \\ \widehat{\beta_1} &= \frac{\boldsymbol{\mu}'(\boldsymbol{Y} - \overline{Y}\boldsymbol{E})}{\boldsymbol{\mu}'(\boldsymbol{\mu} - \overline{\mu}\boldsymbol{E})} \quad (\overline{Y} = \frac{1}{t}\sum_{i=1}^{t}Y_i, \quad \overline{\mu} = \frac{1}{t}\sum_{i=1}^{t}\mu_i) \\ \widehat{\boldsymbol{\mu}} &= (N\boldsymbol{\Sigma}^{-1} + \frac{\beta_1^2}{\sigma^2}\boldsymbol{I})^{-1}(\boldsymbol{\Sigma}^{-1}\sum_{i=1}^{N}a_i(\boldsymbol{X}_i - \boldsymbol{E}b_i) + \frac{\beta_1}{\sigma^2}\boldsymbol{Y} - \frac{\beta_1\beta_0}{\sigma^2}\boldsymbol{E}) \end{aligned}$$

We set initial values and then iterate until convergence to obtain the final solutions.

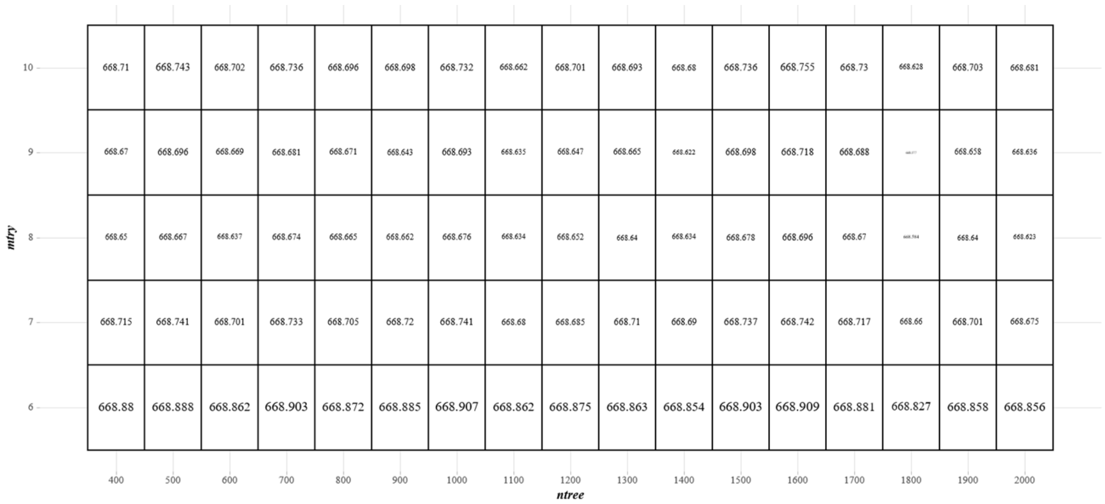

Figure S1. Training performance of random forest models with different parameters.

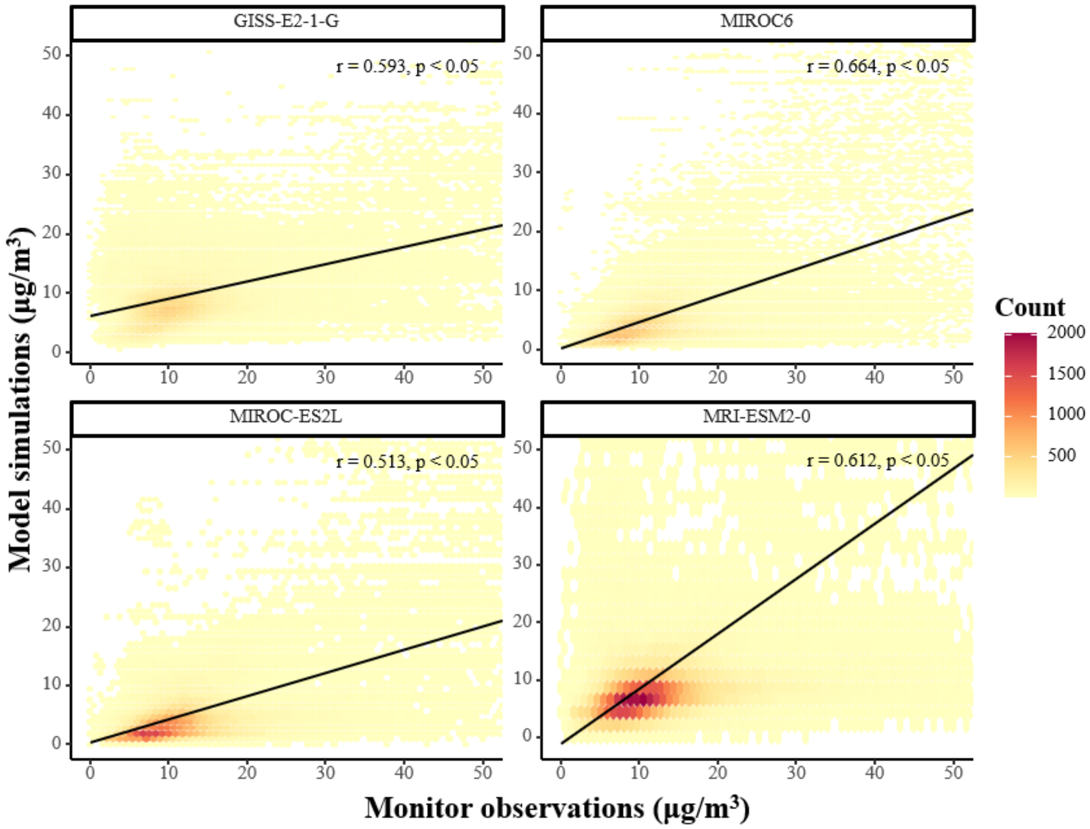

Figure S2. Correlations between the average of ESM-specific simulations and monitoring observations.

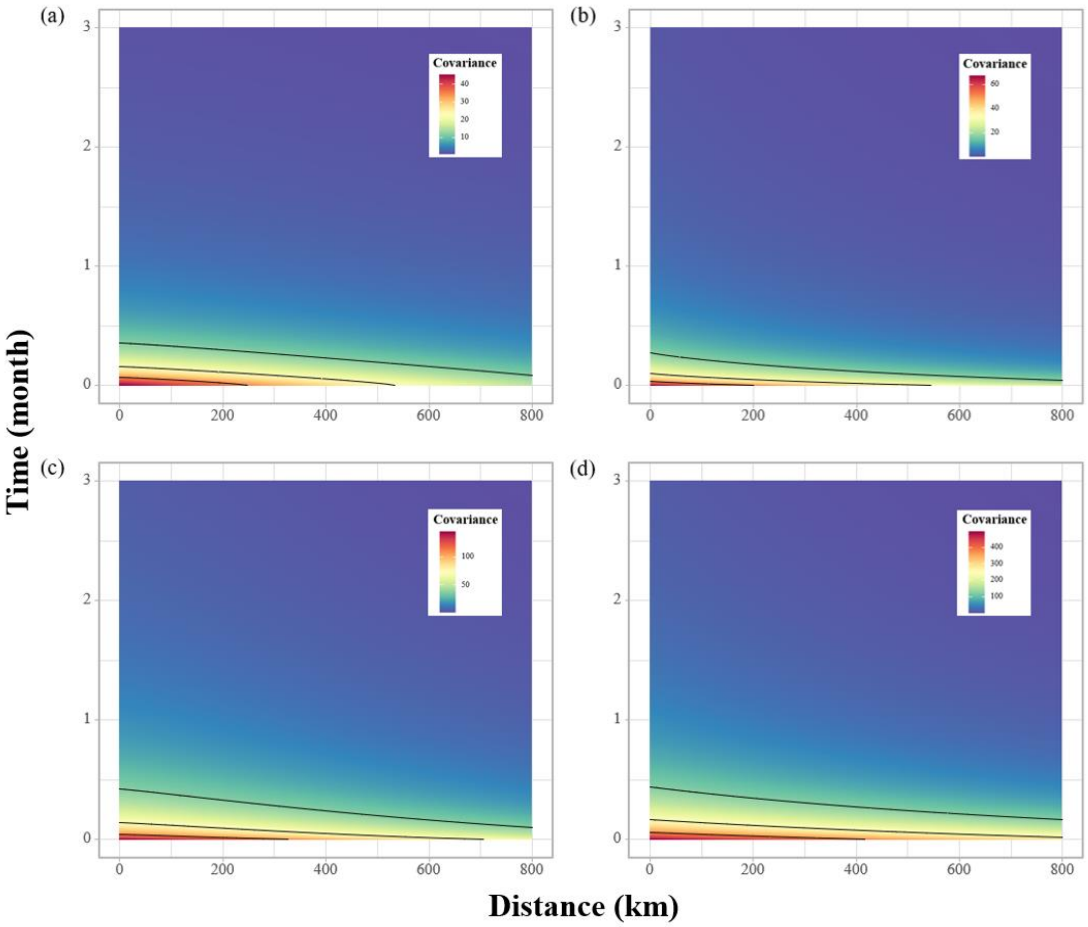

Figure S3. Spatiotemporal covariance of the measurement errors for ESM-specific simulations. Covariance is shown for (a) GISS-E2-1-G, (b) MIROC6, (c) MIROC-ES2L, and (d) MRI-ESM2-0.

Table S1. Selected models in CMIP6.

Institution	Model name	Grid size	Variant name
NASA Goddard Institute for Space Studies, USA	Goddard Institute for Space Studies Model E2.1G (GISS-E2-1-G)	144 × 90	r1i1p3f1<br>r2i1p3f1<br>r3i1p3f1<br>r4i1p3f1<br>r5i1p3f1<br>r6i1p3f1<br>r7i1p3f1<br>r8i1p3f1<br>r9i1p3f1<br>r10i1p3f1
	Goddard Institute for Space Studies Model E2.1H (GISS-E2-1-H)	144 × 90	r3i1p3f1<br>r4i1p3f1<br>r5i1p3f1
	Goddard Institute for Space Studies Model E2.2G (GISS-E2-2-G)	144 × 90	r1i1p3f1<br>r2i1p3f1<br>r3i1p3f1<br>r4i1p3f1<br>r5i1p3f1
The University of Tokyo, National Institute for Environmental Studies, and Japan Agency for Marine-Earth Science and Technology, Japan	Model for Interdisciplinary Research on Climate version 6 (MIROC6)	256 × 128	r1i1p1f1<br>r2i1p1f1<br>r3i1p1f1<br>r4i1p1f1<br>r5i1p1f1<br>r6i1p1f1<br>r7i1p1f1<br>r8i1p1f1<br>r9i1p1f1<br>r10i1p1f1
	Model for Interdisciplinary Research on Climate, Earth System version 2 for High-Resolution simulations (MIROC-ES2H)	256 × 128	r1i1p4f2<br>r2i1p4f2<br>r3i1p4f2
	Model for Interdisciplinary Research on Climate, Earth System version 2 for Long-term simulations (MIROC-ES2L)	128 × 64	r1i1p1f2<br>r1i1000p1f2<br>r2i1p1f2<br>r3i1p1f2<br>r4i1p1f2<br>r5i1p1f2<br>r6i1p1f2<br>r7i1p1f2<br>r8i1p1f2

			r9i1p1f2<br>r10i1p1f2<br>r11i1p1f2<br>r12i1p1f2<br>r13i1p1f2<br>r14i1p1f2<br>r15i1p1f2<br>r16i1p1f2<br>r17i1p1f2<br>r18i1p1f2<br>r19i1p1f2<br>r20i1p1f2<br>r21i1p1f2<br>r22i1p1f2<br>r23i1p1f2<br>r24i1p1f2<br>r25i1p1f2<br>r27i1p1f2<br>r28i1p1f2<br>r29i1p1f2<br>r30i1p1f2
Max Planck Institute for Meteorology, University of Hamburg, and German Climate Computing Center, Germany	Max Planck Institute Earth System Model version 1.2 with Hamburg Aerosol Module (MPI-ESM-1-2-HAM)	192 × 96	r1i1p1f1<br>r2i1p1f1<br>r3i1p1f1
Meteorological Research Institute and Japan Meteorological Agency, Japan	Meteorological Research Institute Earth System Model version 2.0 (MRI-ESM2-0)	192 × 96	r1i1p1f1<br>r1i1000p1f1<br>r3i1p1f1<br>r4i1p1f1<br>r5i1p1f1<br>r6i1p1f1<br>r7i1p1f1<br>r8i1p1f1<br>r9i1p1f1<br>r10i1p1f1
Norwegian Climate Center, Norway	Norwegian Earth System Model version 2 with Low-resolution Atmosphere (NorESM2-LM)	144 × 96	r1i1p1f1
	Norwegian Earth System Model version 2 with Medium-resolution	288 × 192	r1i1p1f1

	Atmosphere<br>(NorESM2-MM)	

Table S2. Spearman correlation coefficients and linear regression results between the model simulations and monitoring observations.

Simulation	r	Slope	Intercept	Residual standard error (RSE)
**GISS-E2-1-G**				
r1i1p3f1	0.583	0.298	6.092	6.621
r2i1p3f1	0.562	0.276	6.283	6.472
r3i1p3f1	0.575	0.305	6.174	6.907
r4i1p3f1	0.577	0.293	6.056	6.613
r5i1p3f1	0.572	0.294	6.074	6.718
r6i1p3f1	0.532	0.287	6.513	7.283
r7i1p3f1	0.563	0.284	6.175	6.660
r8i1p3f1	0.564	0.289	6.284	6.752
r9i1p3f1	0.563	0.296	6.130	6.933
r10i1p3f1	0.565	0.288	6.188	6.704
**GISS-E2-1-H**				
r3i1p3f1	0.580	0.300	6.108	6.704
r4i1p3f1	0.582	0.305	6.044	6.795
r5i1p3f1	0.592	0.301	5.859	6.527
**GISS-E2-2-G**				
r1i1p3f1	0.592	0.357	7.379	7.748
r2i1p3f1	0.576	0.367	7.677	8.289
r3i1p3f1	0.585	0.348	7.594	7.684
r4i1p3f1	0.583	0.382	7.405	8.496
r5i1p3f1	0.599	0.357	7.413	7.612
**MIROC6**				
r1i1p1f1	0.648	0.442	0.264	8.265
r2i1p1f1	0.652	0.432	0.370	8.018
r3i1p1f1	0.656	0.439	0.245	8.060
r4i1p1f1	0.654	0.451	0.144	8.322
r5i1p1f1	0.656	0.447	0.299	8.208
r6i1p1f1	0.655	0.443	0.258	8.146
r7i1p1f1	0.677	0.480	-0.176	8.326
r8i1p1f1	0.647	0.431	0.397	8.094
r9i1p1f1	0.658	0.454	0.082	8.294
r10i1p1f1	0.673	0.458	0.070	8.045
**MIROC-ES2H**				
r1i1p4f2	0.639	0.486	0.623	9.331
r2i1p4f2	0.625	0.464	0.999	9.246
r3i1p4f2	0.638	0.478	0.802	9.198
**MIROC-ES2L**				
r1i1p1f2	0.468	0.376	0.450	11.323
r1i1000p1f2	0.501	0.359	0.606	9.888
r2i1p1f2	0.484	0.393	0.281	11.325

r3i1p1f2	0.458	0.387	0.412	11.985
r4i1p1f2	0.435	0.413	0.209	13.604
r5i1p1f2	0.433	0.416	0.245	13.808
r6i1p1f2	0.461	0.401	0.278	12.325
r7i1p1f2	0.454	0.422	0.045	13.204
r8i1p1f2	0.453	0.383	0.411	12.034
r9i1p1f2	0.447	0.419	0.136	13.344
r10i1p1f2	0.502	0.369	0.529	10.155
r11i1p1f2	0.497	0.404	0.182	11.254
r12i1p1f2	0.469	0.382	0.418	11.464
r13i1p1f2	0.424	0.369	0.680	12.566
r14i1p1f2	0.452	0.373	0.551	11.751
r15i1p1f2	0.506	0.379	0.452	10.302
r16i1p1f2	0.438	0.400	0.276	13.091
r17i1p1f2	0.454	0.391	0.370	12.245
r18i1p1f2	0.454	0.394	0.422	12.313
r19i1p1f2	0.458	0.380	0.522	11.761
r20i1p1f2	0.526	0.361	0.516	9.303
r21i1p1f2	0.465	0.363	0.674	11.028
r22i1p1f2	0.429	0.410	0.280	13.741
r23i1p1f2	0.486	0.399	0.183	11.419
r24i1p1f2	0.399	0.395	0.433	14.486
r25i1p1f2	0.482	0.370	0.482	10.732
r27i1p1f2	0.503	0.430	-0.134	11.787
r28i1p1f2	0.440	0.425	-0.009	13.808
r29i1p1f2	0.431	0.390	0.388	13.005
r30i1p1f2	0.439	0.397	0.375	12.989
**MPI-ESM-1-2-HAM**				
r1i1p1f1	0.576	0.536	0.887	12.112
r2i1p1f1	0.611	0.558	0.207	11.530
r3i1p1f1	0.515	0.584	0.561	15.500
**MRI-ESM2-0**				
r1i1p1f1	0.579	0.982	-1.746	22.068
r1i1000p1f1	0.552	0.823	0.352	19.801
r3i1p1f1	0.603	0.852	-0.267	17.995
r4i1p1f1	0.544	1.005	-1.408	24.708
r5i1p1f1	0.584	0.948	-1.275	21.030
r6i1p1f1	0.557	1.124	-2.687	26.721
r7i1p1f1	0.555	1.064	-2.640	25.473
r8i1p1f1	0.566	0.826	0.133	19.171
r9i1p1f1	0.584	0.982	-1.534	21.744
r10i1p1f1	0.553	0.976	-1.303	23.458
**NorESM2-LM**				
r1i1p1f1	0.543	0.251	4.354	6.179

**NorESM2-MM**				
r1i1p1f1	0.628	0.355	3.374	6.999
**Average**	0.704	0.462	1.704	7.439

Table S3. Overview of the DHS datasets.

Survey	Type	Phase
Albania 2008-09	Standard DHS	DHS-V
Albania 2017-18	Standard DHS	DHS-VII
Angola 2015-16	Standard DHS	DHS-VII
Armenia 2010	Standard DHS	DHS-VI
Armenia 2015-16	Standard DHS	DHS-VII
Bangladesh 2017-18	Standard DHS	DHS-VII
Benin 2001	Standard DHS	DHS-IV
Benin 2011-12	Standard DHS	DHS-VI
Benin 2017-18	Standard DHS	DHS-VII
Burkina Faso 2003	Standard DHS	DHS-IV
Burkina Faso 2010	Standard DHS	DHS-VI
Burundi 2010	Standard DHS	DHS-VI
Burundi 2016-17	Standard DHS	DHS-VII
Cambodia 2005	Standard DHS	DHS-V
Cambodia 2010	Standard DHS	DHS-VI
Cambodia 2014	Standard DHS	DHS-VII
Cameroon 2004	Standard DHS	DHS-V
Cameroon 2011	Standard DHS	DHS-VI
Cameroon 2018	Standard DHS	DHS-VII
Chad 2014-15	Standard DHS	DHS-VII
Colombia 2010	Standard DHS	DHS-VI
Comoros 2012	Standard DHS	DHS-VI
Congo Democratic Republic 2007	Standard DHS	DHS-V
Congo Democratic Republic 2013-14	Standard DHS	DHS-VI
Cote d'Ivoire 2011-12	Standard DHS	DHS-VI
Dominican Republic 2013	Special DHS	DHS-VI
Dominican Republic 2007	Standard DHS	DHS-V
Dominican Republic 2013	Standard DHS	DHS-VI
Egypt 2005	Standard DHS	DHS-V
Egypt 2008	Standard DHS	DHS-V
Egypt 2014	Standard DHS	DHS-VII
Eswatini 2006-07	Standard DHS	DHS-V
Ethiopia 2011	Standard DHS	DHS-VI
Ethiopia 2016	Standard DHS	DHS-VII
Gabon 2012	Standard DHS	DHS-VI
Ghana 2003	Standard DHS	DHS-IV

Ghana 2008	Standard DHS	DHS-V
Ghana 2014	Standard DHS	DHS-VII
Guatemala 2014-15	Standard DHS	DHS-VII
Guinea 2005	Standard DHS	DHS-V
Guinea 2012	Standard DHS	DHS-VI
Guinea 2018	Standard DHS	DHS-VII
Guyana 2009	Standard DHS	DHS-VI
Haiti 2005-2006	Standard DHS	DHS-V
Haiti 2012	Standard DHS	DHS-VI
Haiti 2016-17	Standard DHS	DHS-VII
Honduras 2011-12	Standard DHS	DHS-VI
India 2015-16	Standard DHS	DHS-VII
India 2019-21	Standard DHS	DHS-VII
Indonesia 2002-03	Standard DHS	DHS-IV
Jordan 2002	Standard DHS	DHS-IV
Jordan 2007	Standard DHS	DHS-V
Jordan 2012	Standard DHS	DHS-VI
Jordan 2017-18	Standard DHS	DHS-VII
Kenya 2003	Standard DHS	DHS-IV
Kenya 2008-09	Standard DHS	DHS-V
Kenya 2014	Standard DHS	DHS-VII
Kyrgyz Republic 2012	Standard DHS	DHS-VI
Lesotho 2004	Standard DHS	DHS-V
Lesotho 2009	Standard DHS	DHS-VI
Lesotho 2014	Standard DHS	DHS-VII
Liberia 2007	Standard DHS	DHS-V
Liberia 2013	Standard DHS	DHS-VI
Liberia 2019-20	Standard DHS	DHS-VIII
Madagascar 2008-09	Standard DHS	DHS-V
Malawi 2000	Standard DHS	DHS-IV
Malawi 2004	Standard DHS	DHS-V
Malawi 2010	Standard DHS	DHS-VI
Malawi 2015-16	Standard DHS	DHS-VII
Mali 2001	Standard DHS	DHS-IV
Mali 2006	Standard DHS	DHS-V
Mali 2012-13	Standard DHS	DHS-VI
Mali 2018	Standard DHS	DHS-VII
Moldova 2005	Standard DHS	DHS-V

Morocco 2003-04	Standard DHS	DHS-IV
Mozambique 2011	Standard DHS	DHS-VI
Myanmar 2015-16	Standard DHS	DHS-VII
Namibia 2000	Standard DHS	DHS-IV
Namibia 2006-07	Standard DHS	DHS-V
Namibia 2013	Standard DHS	DHS-VI
Nepal 2006	Standard DHS	DHS-V
Nepal 2011	Standard DHS	DHS-VI
Nepal 2016	Standard DHS	DHS-VII
Niger 2012	Standard DHS	DHS-VI
Nigeria 2003	Standard DHS	DHS-IV
Nigeria 2008	Standard DHS	DHS-V
Nigeria 2013	Standard DHS	DHS-VI
Nigeria 2018	Standard DHS	DHS-VII
Pakistan 2006-07	Standard DHS	DHS-V
Pakistan 2017-18	Standard DHS	DHS-VII
Peru 2000	Continuous DHS	DHS-IV
Peru 2009	Continuous DHS	DHS-VI
Philippines 2003	Standard DHS	DHS-IV
Philippines 2008	Standard DHS	DHS-V
Philippines 2017	Standard DHS	DHS-VII
Rwanda 2005	Standard DHS	DHS-V
Rwanda 2010	Standard DHS	DHS-VI
Rwanda 2014-15	Standard DHS	DHS-VII
Rwanda 2019-20	Standard DHS	DHS-VIII
Senegal 2012-13	Continuous DHS	DHS-VI
Senegal 2014-6R	Continuous DHS	DHS-VII
Senegal 2014-70	Continuous DHS	DHS-VII
Senegal 2015	Continuous DHS	DHS-VII
Senegal 2016-71	Continuous DHS	DHS-VII
Senegal 2017	Continuous DHS	DHS-VII
Senegal 2018	Continuous DHS	DHS-VII
Senegal 2019	Continuous DHS	DHS-VIII
Senegal 2005	Standard DHS	DHS-V
Senegal 2010-11	Standard DHS	DHS-VI
Sierra Leone 2008	Standard DHS	DHS-V
Sierra Leone 2013	Standard DHS	DHS-VI
Sierra Leone 2019	Standard DHS	DHS-VIII

Tajikistan 2012	Standard DHS	DHS-VI
Tajikistan 2017	Standard DHS	DHS-VII
Tanzania 2010	Standard DHS	DHS-VI
Tanzania 2015-16	Standard DHS	DHS-VII
Timor-Leste 2009-10	Standard DHS	DHS-VI
Timor-Leste 2016	Standard DHS	DHS-VII
Togo 2013-14	Standard DHS	DHS-VI
Uganda 2000-01	Standard DHS	DHS-IV
Uganda 2006	Standard DHS	DHS-V
Uganda 2011	Standard DHS	DHS-VI
Uganda 2016	Standard DHS	DHS-VII
Zambia 2007	Standard DHS	DHS-V
Zambia 2013-14	Standard DHS	DHS-VI
Zambia 2018	Standard DHS	DHS-VII
Zimbabwe 2005-06	Standard DHS	DHS-V
Zimbabwe 2010-11	Standard DHS	DHS-VI
Zimbabwe 2015	Standard DHS	DHS-VII
